# Trends in Pricing and Out-of-Pocket Spending on Entecavir Among Commercially Insured Patients, 2014-2018

**DOI:** 10.1001/jamanetworkopen.2021.44521

**Published:** 2022-01-21

**Authors:** Jonathan D. Alpern, Heesoo Joo, Ben Link, Antonio Ciaccia, William M. Stauffer, Nathan C. Bahr, Thomas M. Leventhal

**Affiliations:** 1 HealthPartners Infectious Diseases, St Paul, Minnesota; 2 Division of Global Migration and Quarantine, Centers for Disease Control and Prevention, Atlanta, Georgia; 3 46brooklyn Research, Dayton, Ohio; 4 Center for Global Health and Social Responsibility, School of Public Health, University of Minnesota, Minneapolis; 5 Division of Infectious Diseases, Department of Medicine, University of Kansas Medical Center, Kansas City; 6 Division of Gastroenterology, Hepatology and Nutrition, Department of Medicine, University of Minnesota, Minneapolis

## Abstract

This cross-sectional study of health claims from patients with private insurance examines trends in the cost of entecavir prescribed for chronic hepatitis B treatment.

## Introduction

Chronic hepatitis B (CHB) causes significant liver-related morbidity and mortality. Treatment of CHB is cost-effective in the US; however, high out-of-pocket spending on first-line therapy may be a treatment barrier.

Entecavir, a generic drug that is one of the first-line agents used for treatment of CHB, has had a steep decline in the average price that pharmacies pay for the drug (ie, national average drug acquisition cost [NADAC]) because of manufacturer competition. Yet, the list price—which correlates with out-of-pocket spending—has remained high. We assessed trends in the number of manufacturers, average wholesale price, NADAC, and out-of-pocket spending for entecavir among a commercially insured population with CHB between 2014 and 2018.

## Methods

For this cross-sectional study, yearly NADAC prices of entecavir 0.5-mg tablets were obtained from Medicaid’s publicly available NADAC database for the last week of December 2014 through December 2018.^1^ We calculated yearly average wholesale price based upon a weighted average calculation of Medicaid expenditures and average wholesale price derived from a criterion standard drug database (ProspectoRx).^2^ We used the US Food and Drug Administration’s Orange Book to determine the yearly number of drug manufacturers. We analyzed a commercial database of health claims using cloud-based analytic interface (IBM Corp) from 2014 (the year of entecavir generic entry) to 2018 (the most recent data available). We limited our analysis to continuously enrolled members with private insurance and with a CHB diagnosis code associated with a visit within 2 years of the analysis period and a claim for entecavir 0.5- or 1-mg tablets (eAppendix in the Supplement). We obtained utilization and fill data for entecavir and calculated mean number of fills per member, mean number of days of supply per member, and mean annual out-of-pocket spending, as well as total spending per member, fill, and 30-day supply stratified by use of a high-deductible health plan. We followed the Strengthening the Reporting of Observational Studies in Epidemiology (STROBE) reporting guideline for observational studies. The protocol for this study was reviewed by the US Centers for Disease Control and Prevention and was granted exempt status. The study used data from a deidentified database. All results are presented in aggregate form, and specific patients were not identified; thus, informed consent was not required in accordance with 45 CFR §46. Data were analyzed using Stata SE version 16.1 (StataCorp).

## Results

Between 2014 and 2018, there were over 1000 annual entecavir fills, and a mean (SD) of 6.7 (3.8) annual fills per member. As the number of entecavir manufacturers increased from 1 to 11, the NADAC decreased from $30.12 to $1.93 per 0.5-mg tablet. The average wholesale price remained constant at $44.43. Among commercially insured members, mean (SD) out-of-pocket spending per 30-day supply of generic entecavir was $41 ($81) in 2014 and $52 ($97) in 2018. Mean (SD) out-of-pocket spending per 30-day supply of brand name entecavir was $118 ($180) in 2014 and $165 ($178) in 2018. Among members with a high-deductible health plan, mean (SD) out-of-pocket spending per 30-day supply of generic entecavir was $103 ($167) in 2014 and $133 ($122) in 2018. Mean (SD) total spending per 30-day supply of generic entecavir was $981 ($154) in 2014 and $591 ($332) in 2018 (Table). Trends in the number of manufacturers, NADAC, average wholesale price, and out-of-pocket spending per 30-day supply of generic entecavir are shown in the Figure.

**Table.** Annual Total and OOP Spending on Entecavir, 2014-2018

Characteristic	Spending per year, mean (SD), $				
	2014	2015	2016	2017	2018
**Overall**					
Total patients filling ≥1 prescription, No.	1783	1646	1716	1649	1406
Fills per patient, mean (SD), No.	6.6 (3.8)	6.7 (3.8)	6.7 (3.7)	6.7 (3.9)	6.7 (3.9)
Supply per patient, mean (SD), d	293 (110)	299 (111)	298 (103)	297 (106)	298 (104)
OOP spending per year					
Mean per patient	721 (1028)	679 (1160)	732 (1183)	638 (1041)	622 (998)
Mean per fill	136 (253)	123 (246)	128 (237)	116 (238)	116 (220)
Mean per 30-d supply	81 (134)	73 (132)	78 (131)	69 (122)	67 (116)
HDHP	232 (218)	203 (201)	216 (167)	194 (190)	163 (138)
Non-HDHP	66 (113)	60 (114)	63 (117)	54 (102)	54 (106)
Total spending per year^a^					
Mean per patient	10 913 (4225)	10 073 (4267)	8794 (4119)	7410 (4789)	6720 (4801)
Mean per fill	2020 (1051)	1819 (944)	1587 (904)	1342 (974)	1226 (989)
Mean per 30-d supply	1121 (183)	1013 (247)	890 (271)	750 (373)	681 (396)
HDHP	1143 (272)	960 (204)	883 (269)	661 (382)	628 (413)
Non-HDHP	1119 (172)	1019 (251)	891 (272)	761 (371)	688 (393)
**Brand drug only**					
Total patients filling ≥1 prescription, No.	733	361	276	205	159
Fills per patient, mean (SD), No.	6.8 (4.1)	8.3 (3.9)	8.4 (3.9)	8.3 (4.1)	7.9 (4.1)
Supply per patient, mean (SD), d	267 (115)	312 (96)	306 (94)	310 (94)	307 (93)
OOP spending per year					
Mean per patient	931 (1195)	1373 (1496)	1607 (1457)	1596 (1478)	1534 (1350)
Mean per fill	174 (319)	210 (294)	237 (312)	252 (382)	257 (376)
Mean per 30-d supply	118 (180)	139 (159)	167 (161)	162 (173)	165 (178)
HDHP	309 (277)	260 (139)	304 (198)	316 (249)	278 (130)
Non-HDHP	99 (155)	129 (157)	146 (145)	136 (141)	140 (178)
Total spending per year^a^					
Mean per patient	10 345 (4695)	12 304 (4100)	12 386 (4445)	13 828 (4562)	13 467 (4527)
Mean per fill	1871 (1035)	1766 (945)	1748 (938)	2050 (1152)	2146 (1225)
Mean per 30-d supply	1164 (183)	1186 (156)	1203 (212)	1330 (176)	1313 (217)
HDHP	1174 (93)	1073 (245)	1057 (296)	1228 (227)	1272 (240)
Non-HDHP	1163 (189)	1195 (144)	1225 (188)	1347 (160)	1322 (211)
**Generic only**					
Total patients filling ≥1 prescription, No.	75	1117	1294	1386	1218
Fills per patient, mean (SD), No.	1.8 (0.9)	5.9 (3.6)	6.2 (3.6)	6.4 (3.8)	6.5 (3.8)
Supply per patient, mean (SD), d	76 (40)	291 (117)	293 (106)	293 (109)	297 (106)
OOP spending per year					
Mean per patient	81 (159)	405 (863)	495 (981)	470 (849)	476 (838)
Mean per fill	49 (85)	92 (228)	100 (207)	93 (202)	95 (177)
Mean per 30-d supply	41 (81)	49 (114)	55 (111)	53 (105)	52 (97)
HDHP	103 (167)	191 (218)	185 (137)	168 (165)	133 (122)
Non-HDHP	31 (54)	34 (84)	42 (98)	40 (87)	42 (88)
Total spending per year^a^					
Mean per patient	2439 (1251)	9120 (4080)	7854 (3611)	6332 (3989)	5746 (4045)
Mean per fill	1498 (820)	1836 (934)	1562 (903)	1229 (900)	1102 (891)
Mean per 30-d supply	981 (154)	949 (233)	816 (236)	656 (312)	591 (332)
HDHP	1065 (104)	908 (182)	823 (239)	538 (290)	495 (309)
Non-HDHP	968 (156)	953 (238)	815 (236)	669 (311)	603 (333)

Abbreviations: HDHP, high-deductible health plan; OOP, out-of-pocket.

^a^ Total spending refers to the sum of OOP and insurance spending.

**Figure.**
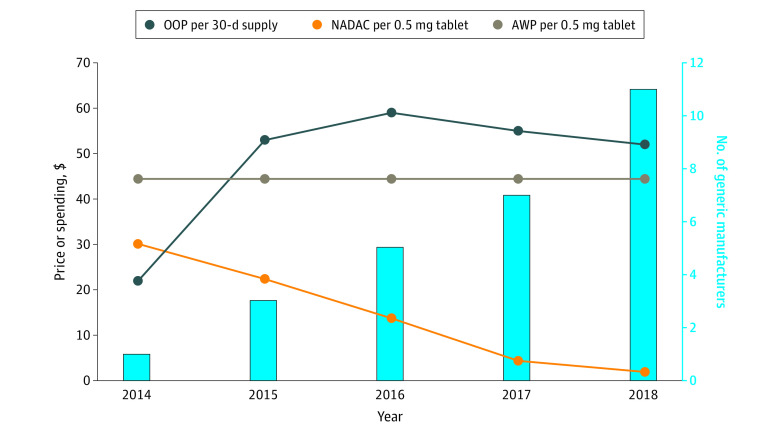
National Average Drug Acquisition Cost (NADAC), Average Wholesale Price (AWP), Out-of-Pocket (OOP) Spending per 30-Day Supply of Generic Entecavir, and Numbers of Generic Entecavir Manufacturers in the US, 2014-2018

## Discussion

Out-of-pocket spending on generic entecavir increased between 2014 and 2016 and remained elevated through 2018 despite robust generic competition and a marked decline in the price that pharmacies paid for entecavir. In 2018, despite 11 approved manufacturers, patients on high-deductible health plans spent a mean of $133 per 30-day supply—a threshold associated with a higher than 50% rate of prescription abandonment.^3^ The artificially high average wholesale price for entecavir is a likely driver of such high out-of-pocket spending, as drugs are often paid for based on a discount of average wholesale price, benefiting supply chain intermediaries—such as pharmaceutical benefit managers and wholesalers—while contributing to drug price inflation.^4^ In 2017, pharmaceutical benefit managers’ so-called spread pricing caused Indiana Medicaid to spend over $800 for a 30-day supply of entecavir that cost pharmacies under $140.^5^ Our findings warrant further investigation into the reasons for high out-of-pocket spending on entecavir, and how out-of-pocket spending may factor into prescription abandonment among persons living with CHB—a population predominantly born outside the US and disproportionately affected by the social determinants of health.^6^

Limitations to this study included lack of health plan rebates or patient coupons in spending calculations. Additionally, we did not account for other strategies, such as pill splitting of 1-mg tablets, that could lower out-of-pocket spending. Our findings highlight the need for policies that improve transparency around generic drug financing and pharmaceutical benefit manager practices.

## Notes

Supplement. **eAppendix.** Trends in Pricing and Out-of-Pocket Spending on Entecavir Among Commercially Insured Patients, 2014-2018
