# Error in Figure

**DOI:** 10.1001/jamanetworkopen.2022.5261

**Published:** 2022-03-10

In the Research Letter titled “Trends in Pricing and Out-of-Pocket Spending on Entecavir Among Commercially Insured Patients, 2014-2018”,^1^ published January 21, 2022, an older version of the Figure was inadvertently published. The Figure included data points for out-of-pocket spending that incorrectly included a denominator of patients taking both brand name and generic entecavir, rather than generic only. This article has been corrected.^1^
