# Supplementary material for: Trends in Pricing and Out-of-Pocket Spending on Entecavir Among Commercially Insured Patients, 2014-2018
Source: JAMA Netw Open. 2022 Jan 21;5(1):e2144521. doi: 10.1001/jamanetworkopen.2021.44521 (PMC8783269; doi:10.1001/jamanetworkopen.2021.44521)
Supplement: Supplement. — eAppendix. Trends in Pricing and Out-of-Pocket Spending on Entecavir Among Commercially Insured Patients, 2014-2018 [file jamanetwopen-e2144521-s001.pdf]

Supplemental Online Content

Alpern JD, Joo H, Link B, et al. Trends in pricing and out-of-pocket spending on entecavir among commercially insured patients, 2014-2018. *JAMA Netw Open.* 2022;5(1):e2144521. doi:10.1001/jamanetworkopen.2021.44521

eAppendix. Trends in Pricing and Out-of-Pocket Spending on Entecavir Among Commercially Insured Patients, 2014-2018

This supplemental material has been provided by the authors to give readers additional information about their work.

© 2022 Alpern JD et al. *JAMA Network Open.*

eAppendix. Trends in Pricing and Out-of-Pocket Spending on Entecavir among Commercially Insured Patients, 2014-2018

Patients with a diagnosis of hepatitis B in the IBM MarketScan® Commercial Database were selected using the IBM MarketScan® Treatment Pathways (MarketScan 100% 6 plus years – 1/1/2013 through 7/31/2020).[1, 2] We identified all patients with a diagnosis of hepatitis B (International Classification of Diseases, Ninth Revision, Clinical Modification [ICD-9-CM] diagnosis codes 070.20, 070.21, 070.22, 070.23, 070.30, 070.31, 070.32, and 070.33 for data prior to September 30, 2015, and ICD-10-CM diagnosis codes B160, B161, B162, B169, B16, B170, B180. B181, B1910, B1911, and B191 after September 30, 2015). For each year, we selected patients who had diagnosis codes in the previous or current years. For instance, we defined the patients in 2014 using those who reported hepatitis B diagnosis codes between January 1, 2013 and December 31, 2014 because those who have chronic hepatitis B may not have inpatient or outpatient visits every year but continuously use prescription fills to manage their condition.

We excluded those with a diagnosis of human immunodeficiency virus (HIV) using ICD-9-CM codes 042 or 079.53 and ICD-10-CM codes B20, Z21, or B9735. We restricted the sample to include patients enrolled in private insurance only, excluding those who were covered by employer-sponsored Medicare Supplemental plans as Medicare payments were not included in the datasets. Patients needed to be continuously enrolled to a non-capitated insurance plan in each year. Among members continuously enrolled to a non-capitated insurance, those who utilized entecavir 0.5 or 1 mg tablets were included in the analyses. Although tenofovir disoproxil fumarate (TDF) and tenofovir alafenamide (TAF) are also first-line drugs used to treat

© 2022 Alpern JD et al. *JAMA Network Open.*

chronic hepatitis B, we did not analyze TDF because of limited NADAC data available (2017-2018) or TAF because it is a brand-name drug. We examined those who utilized generic or brand drugs and separately examined those who utilized generic and those who utilized brand drugs. We defined those who utilized generic or brand name drugs by using ingredient name, entecavir. For those who utilized generic entecavir, we used product name, Entecavir and Entecavir Avpak. We excluded those who reported total drug payments, including both out-of-pocket and insurance drug payments, below the 1st or above the 99th percentile to rule out outliers.

This is a descriptive study, calculating means of: number of fills, days of supply, and out-of-pocket spending per patient per year between 2014 and 2018. Thus, this study does not demonstrate any causality. Mean number of fills per patient per year was the calculated average of annual numbers of entecavir prescription fills for each patient. Those who used entecavir were included in the analysis. Mean number of days of supply per patient per year was the calculated average of annual numbers of days of entecavir supply per patient. Mean out-of-pocket spending for entecavir per patient per year was the calculated average of annual out-of-pocket costs for entecavir per patient. Annual out-of-pocket drug spending per patient would be helpful to understand the annual burden for hepatitis B patients. However, if a patient started new anti-hepatitis B medication during the middle of the year, the annual out-of-pocket spending could be underestimated.

To mitigate potential bias, we estimated mean out-of-pocket spending for entecavir per fill. Out-of-pocket drug spending per fill for each patient was estimated by dividing each patient's annual out-of-pocket drug spending by each patient's annual number of fills. The mean out-of-pocket drug spending per fill reduced the bias caused by the adaptation of new

© 2022 Alpern JD et al. *JAMA Network Open.*

medication. However, when we checked days of supply per fill for each patient, there were variations, which may cause another type of bias.

Thus, we additionally reported mean out-of-pocket drug spending per 30-day supply to reduce the bias caused by variations in days of supply per fill because the most common average period per fill for entecavir, including both generic and brand name drugs, was 30 days. The average 30-day supply per fill was reported by 49% of those patients who utilized entecavir. Next, the average 90-day supply per fill was reported by 37% of those who utilized entecavir. Other periods per fill, which may be a result of patients changing drug filling cycles within a calendar year, were reported by the remaining 14% of those who utilized entecavir. We calculated each patient's daily out-of-pocket drug spending for entecavir and multiplied by 30 to get the drug spending per 30-day supply for each patient. Then, we calculated average of drug spending per 30-day supply for each patient. We also reported total spending, including out-of-pocket spending by patients and payments by insurance.

Reference

1. IBM MarketScan® Commercial Database. www.ibm.com/products/marketscan-research-databases/databases. Accessed September 20, 2021.
2. IBM MarketScan® Treatment Pathways. www.ibm.com/products/marketscan-research-databases/analytic-tools. Accessed September 20, 2021.

© 2022 Alpern JD et al. *JAMA Network Open.*
